# Who is benefiting from the dramatic decline in U.S. cancer mortality? Place-based evidence of disparities in rates of improvement

**DOI:** 10.1038/s41416-026-03339-8

**Published:** 2026-03-30

**Authors:** Arthur G. Cosby, Viswadeep Lebakula, Karissa Bergene, Gina Rico Mendez, Mackenzie Bumgarner, Alina Peluso

**Affiliations:** 1 Social Science Research Center, Mississippi State University, Starkville, MS USA; 2 Geospatial Science and Human Security Division, National Security Sciences Directorate, Oak Ridge National Laboratory, Oak Ridge, TN USA; 3 Office of Strategic Operations, William Carey University, Hattiesburg, MS USA; 4 Computational Sciences and Engineering Division, Computing and Computational Sciences Directorate, Oak Ridge National Laboratory, Oak Ridge, TN USA

**Keywords:** Sociology, Developing world, Risk factors

## Abstract

**Background:**

After decades of increasing cancer mortality, U.S. rates declined from 1991 to 2019, a 32% decrease. we investigated rates of cancer mortality improvement across 2954 counties and selected characteristics associated with mortality improvements.

**Methods:**

Data was 21,381,009 county-level neoplasm deaths gleaned from death certificates via CDC WONDER. Analytical techniques included GIS and Moran’s I, OLS, GWR models, and trend comparisons.

**Results:**

Counties with the greatest improvement (reduction) in cancer mortality tended to be coastal, higher-income, metropolitan locations. OLS model (R^2^ = 0.65) indicated that greatest improvements were observed in counties with higher initial mortality ($\beta =.32$) closely followed by percent urban ($\beta =.31$) and median household income ($\beta =.16$). Whereas percent Black residents ($\beta =-.06$), and percent with education beyond high school ($\beta =-.10$) was less associated on outcomes. Highest income counties were the first to experience improvement in cancer mortality, the highest rates of mortality decline, and the greatest reduction in excess deaths.

**Discussion:**

Even though there was significant improvement in cancer mortality nationally, there were variations in the degree of improvement linked to county location, income, and urbanisation. These results underlie the need to expand place-based initiatives designed to advance cancer health and more equitable improvements in cancer mortality outcomes.

## Introduction

The year 1991 marked a watershed in United States (U.S.) cancer health. That year, cancer mortality rates that had increased throughout the 20^th^ century peaked at 215.1 death per 100,000 [1]. Since then, there was a striking decline to a rate of 146.0 in 2019, a 32% improvement in cancer mortality [1]. Declining death rates from lung and bronchus, breast, prostate, and colorectum cancers were major contributors to improving cancer mortality [2–7]. This resulted in over three million fewer cancer deaths than if cancer mortality had remained at 1991 levels. This is a major accomplishment in national health that enhances not both longevity and wellbeing of the nation [8]. However, in a complex, diverse country, it is unlikely that all segments of the population will benefit equally during a major societal change such as decline in cancer mortality [9]. It is this aspect of societal change that guides our research to determine who is benefitting from improvement in cancer mortality. We are referring to the “collective who” of people living in various counties and to benefits that are broadly defined. High cancer rates devastates families and communities emotionally and economically, and impact human and social capital. When cancer mortality declines, there are substantial benefits beyond fewer deaths.

It is useful to distinguish three dimensions of place-based mortality disparities. The first frequently utilised dimension compares mortality rates between two or more places at a point in time (e.g., county A has a higher cancer mortality rate than county B in 2019). The second compares mortality rates between places at several points in time to determine temporal shifts in the magnitude of mortality disparities (e.g., the disparities in cancer rates between county A and B are increasing over time). The third estimates the rate of change in mortality for each place over one or more periods (e.g., the rate of improvement in cancer mortality is greater for county A than for county B). This third seldom investigated dimension of mortality disparities is the focus and contribution of our research.

In addition, rather than focusing on national rates of mortality improvement, we instead examined yearly percent decline (i.e., improvement) rates in cancer mortality that occurred in each U.S. county from 1981 to 2019 [10, 11]. Contrary to previous studies where mortality counts or rates were typically used, we analysed mortality improvement rates at the county level which more appropriately address the research question. From this more granular geographic perspective, we compared rates of mortality change of places with different social and demographic characteristics. The existence of large variations in improvement between counties implies that national improvement rates are not capturing large geographic and social differences in cancer mortality improvements and thus national rates should be supplemented with more granular data [12, 13].

Our study is informed by the social determinants of health (SDoH) perspective of the World Health Organisation (WHO). SDoH are the nonmedical conditions in which “people are born, grow, live, work and age, and people’s access to power, money, and resources – have a powerful influence on health inequities.” (https://www.who.int/health-topics/social-determinants-of-health) Previous research in the U.S. document the influence of social determinants (e.g., education, income, poverty, race, and urbanisation) on mortality. From this perspective, SDoH function at multiple levels of analysis including individuals, families, groups, and places. Higher income, less poverty, more education, less prejudice and discrimination, and live in urban residents have better health and improved longevity [4, 14–21]. Places with higher incomes often have resources that allow broader access to quality healthcare, healthier environments, enhanced safety, and diminished behavioural risk factors [22–26]. American Cancer Society (ACS) and Centres for Disease Control and Prevention (CDC) have identified factors that underlie decline in cancer mortality. Tobacco use control measures (e.g., smoking cessation, smoke-free place policies, tobacco taxation) have reduced lung cancer and contributed to improvements in many other cancers. Improved screening and detection along with introduction of new treatments has substantially contributed to remission and survivability of cancer patients [2, 17, 27–29]. Therefore, income-linked intervening factors that reduce cancer mortality include tobacco control and smoking cessation, cancer screening, diet, weight control, exercise, limiting exposure to environmental toxins, health policies, and availability of quality healthcare [30, 31]. Furthermore, racial composition can also be considered an important socio-ecological condition. Counties with higher percentages of racial minorities may be subject to prejudice and other stressors that can negatively impact health [22, 32–37]. Urban places often have favourable health and mortality measures in comparison to rural places; this could be attributed to capacity of urban centres to drive innovations in health care, therefore increasing access, and promotion of healthier lifestyles [2, 32, 33, 37–44]. In this study, we analyse and model place-based cancer mortality at the county level to better understand spatial variation of benefits as the U.S. is experiencing an impressive decline in cancer mortality rates.

## Methods

We focused on cancer mortality in the U.S. beginning in 1981 through 2019; a period of significant transition in the nation’s cancer health. This timeframe includes rising national cancer mortality (i.e., 1981 through 1991), peak cancer mortality (i.e., 1991), and declining cancer mortality (i.e., 1991 through 2019). Age-adjusted cancer mortality (AACM) rates were obtained from National Centre for Health Statistics (NCHS) available through CDC WONDER [45]. We combined two databases available in CDC WONDER: Compressed Mortality File (1981–2016) [10] and Underlying Cause of Death File [11] (2017–2019) (refer Supplemental Table 1). From these datasets we selected deaths with International Classification of Diseases (ICD-9 and ICD-10) code for Neoplasms. CDC WONDER also report deaths by age, race, gender, county of residence, and cause of death [10]. The study population consists of reported annual county-level (*n*  =  2954) cancer mortality data from 1981 to 2019 (*n*  =  21,381,009 cancer deaths) for all 50 states. We collapsed county-level mortality data into three-year periods improve reliability estimates of counties, especially for smaller population counties. This method involves a “trade off.” While we lose the ability to track changes on an annual basis, we were able to utilise AACM rates for substantially more counties. By not utilising small area models [46] that include covariates such as county income, education, race, and rural urban status, we were instead able to use these variables to model county-level disparities in cancer mortality reduction (i.e., independent measurement was maintained). Previously, researchers employed similar design decisions of combining multi-year mortality rates for counties with small populations [47, 48]. AACM rates for three-year periods were calculated per 100,000 and adjusted to the year 2000 standard million to account for age structure differences via CDC WONDER analytics. Our primary variable is percent decrease overtime in cancer mortality ($y$) was calculated as percent decrease in AACM rates using the 1981-1983 period as the baseline. The Compressed Mortality File contains 3082 counties, of which we have sufficient cancer data for 2954 counties. CDC WONDER does not provide data for death counts of 10 or less. This resulted in missing data for 128 counties. Deleted counties tended to have small populations. Collectively, they had a combined population of 1,172,268 or 0.36% of total U.S. population in 2019.

We developed quartile GIS maps to visualise the geographic distribution of mortality changes for U.S. counties over the 1981–1983 to 2017–2019. In addition, we calculated and mapped the spatial statistic Local Moran’s I to identify clusters of counties with high and low levels of cancer mortality improvement between these time periods [49]. This algorithm allowed the detection of spatial clusters and outliers which are not easy to detect using global measures [50]. Moran’s I assess the likelihood that a spatial clustering of values occurs by chance, thus providing a measure of spatial autocorrelation. By applying Moran’s I, we identified distinct patterns of cancer mortality change, including clusters of counties with high levels of improvement adjacent to other high-improvement counties (high-highs), as well as clusters of counties with low or no improvement surrounded by other similar counties (low-lows).

Independent variables such as county-level characteristics including race (percent Black), median household income, and education were used to fit multivariate ordinary least square (OLS) regression (Supplemental Table 1). Annual county percent Black population was calculated from 1981 to 2019 using population estimates from the National Cancer Institute - Surveillance, Epidemiology, and End Results (SEER) Programme. We utilised two indicators of county-level socioeconomic status: income and education levels. Income was operationalized as median household income, which was obtained from the Census for years 1979, 1989 and 1999 and from the Agency for Healthcare Research and Quality (AHRA) for 2009 to 2019. Education was defined as percent of adult population with bachelor’s degree or higher, associate’s degree obtained, or some college attended. Education for years 1980, 1990, 2000, and 2014-2018 was obtained from the United States Department of Agriculture (USDA). Our model included a measure degree of urban residence, defined as percent urban, obtained from the Census for years 2000, 2010, and 2020 [51, 52]. It is important to note these variables are calculated as a part of the Census, which are collected every 10 years. For the years between censuses, we incorporated available annual estimates (e.g., race) when provided. For variables with missing data in non-census years, we used linear interpolation to generate annual estimates. For instance, median household income data are available for the years 1979, 1989, 1999, and annually from 2009 to 2019. To fill the gaps for intervening years, we applied linear interpolation to estimate annual values. Subsequently, for each 3-year time period, we calculated averages of the annual values within that interval. For example, the median household income for the 1984–1986 period was computed as the average of the annual measures for 1984, 1985, and 1986. The race measure was calculated as percent Black population. The starting level of AACM for each county in 1981-1983 was included in the model as a control variable.

We modelled county-level rates of cancer mortality improvement by utilising time series analysis when comparing mortality change over a 39-year period. Cancer mortality data were grouped in three-year periods to improve reliability of estimates. We conducted 12 multivariate OLS regression analyses to investigate the association of income, education, race, urban residence, and initial level of cancer mortality (i.e., 1981–1983) on improvement rates in cancer mortality. Conceptually, the OLS models are based on the expectation that favourable SDoH measures at the county-level such as higher median income, higher levels of education, lower percent Black population, and lower percent rural population would be associated with declines in cancer mortality while controlling for the initial level of cancer mortality. Then we evaluated the models over 12 different time periods between 1981 and 2019. This patterning of models allowed us to assess the changing influence of SDoH variables in the model. For example, should the overall power of the OLS models (R^2^) increase at each successive time frame, it would indicate that the association of SDoH with cancer mortality was increasing overtime. To account for variation in size of county population we weighted models by population levels for 2000. To account for variation in county age structure, we utilised AACM rates. We calculated regression models for each of 12 time periods. The model was expressed as:

$$Y=\alpha +{\beta }_{1}{X}_{1}+{\beta }_{2}{X}_{2}+{\beta }_{3}{X}_{3}+{\beta }_{4}{X}_{4}+{\beta }_{5}{X}_{5}+{error}$$

Where X_1_ was median household income, X_2_ was percent bachelor’s degree or higher/associate’s degree obtained/some college attended, X_3_ was percent Black, X_4_ was percent urban, and X_5_ was initial level of cancer mortality (i.e., 1981–1983). Betas ($\beta$_1_ through $\beta$_5_) were standardised parameter estimates for input variables. Coefficients of determination (R^2^) provided an estimate of explanatory power of models. Estimates of 12 OLS models became time series data to evaluate effectiveness of models over time and are interpreted as timewise impacts of social influences on cancer mortality improvement.

In addition to the OLS regression, we analysed the OLS function via Geographically Weighted Regression (GWR) to explore spatially varying relationships between SDoH and cancer mortality improvement [53, 54]. GWR allows for local estimation of the regression coefficients at each geographic unit, considering the spatial dependencies of neighbouring counties. In other words, GWR generates a separate OLS regression equation for each county, which provides an improved local understanding of various county-level measures such as income, education, and race [55, 56]. The coefficients from the GWR model provide a more nuanced understanding of spatial heterogeneity and the localised effects of these social factors. By comparing GWR results to OLS estimates, we were able to assess the influence of geographical context on the relationships between social variables and cancer mortality trends.

## Results

**Fig. 1:**
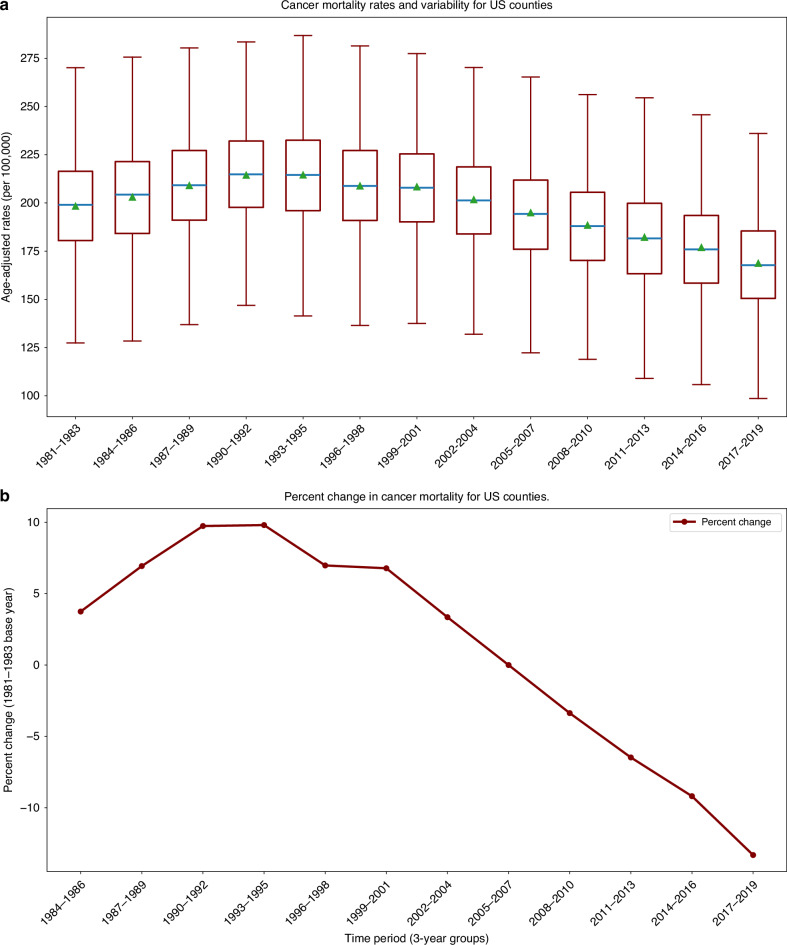
Trends in Place-based Age-adjusted Cancer Mortality Patterns for U.S. Counties: 1981–2019. **a** Boxplot visualisation includes average county age-adjusted mortality rate (green triangle), median (blue line), and the box and whiskers depict the county distribution in quartiles (without outliers). **b** Average percent change in age-adjusted county mortality rates for each three-year period with the 1981–1983 period as the base year.

In Fig. 1a distribution of county-level AACM rates was approximately symmetrical. Mean county rates were initially 198.36 deaths per 100,000 (age-adjusted) in 1981–1983, then increased to 214.53 in 1993–1995, and began to fall to a record low of 168.75 in 2017–2019. Despite this success in reducing cancer mortality at the national level, substantial county-level variability indicated large spatial disparities. In summary, Fig. 1a depicts rapidly improving cancer mortality overall, yet persistent large disparities in mortality rates among counties as the time series evolved.

Degree of county-level benefit was estimated by calculating rate of change in AACM for each county across 12 time periods between 1981–1983 and 2017–2019. The initial 1981–1983 period was used as baseline for mortality change. Hence, we have 2954 change rates for each of 12 time periods for a total of 35,448 different rates of change. This data matrix provides a more comprehensive measurement of spatial and temporal complexities of U.S. cancer mortality than national estimates alone. Average county-level cancer mortality changed in three parts (Fig. 1b). First, county-level cancer rates were increasing prior to 1990–1992 period. Second, there was a turnaround in 1990–1992 where mortality rates leveled off and began to decline. Third, beginning with 1999–2001 period, there was a sharp decline that continued through the most recent data in the 2017–2019 period. The cancer mortality rate peaked at 214.54 in 1993–1995 and declined to 168.75 in 2017–2019. Overall percent decrease during this period was 21.3%.

### The U.S. counties with the greatest improvement in cancer mortality tended to be located along coasts in metropolitan locations

**Fig. 2:**
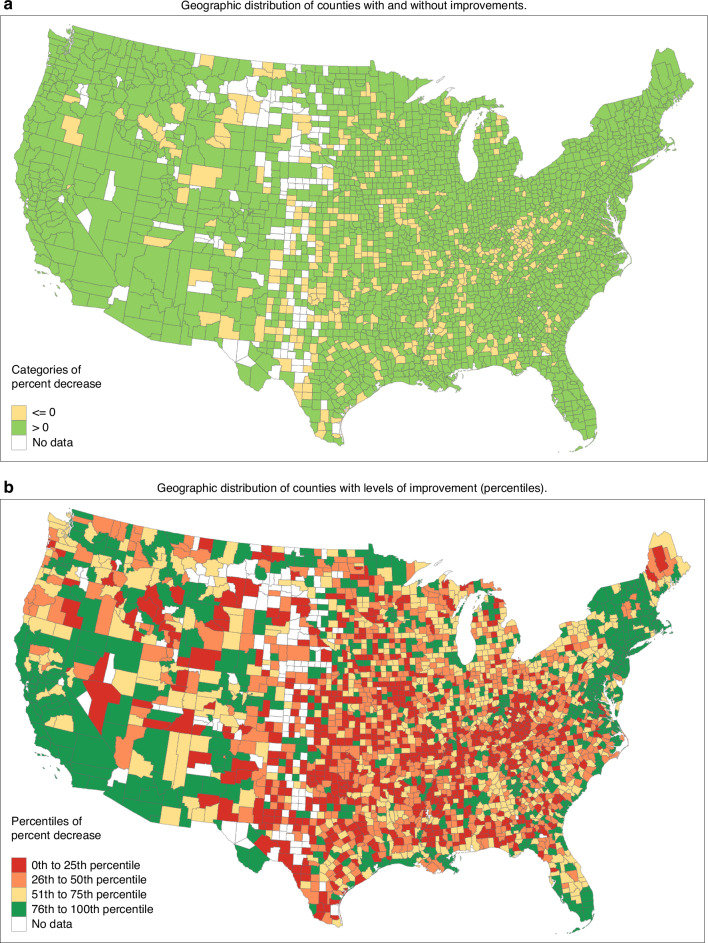
Geographic distribution of percent decrease in age-adjusted cancer mortality rates for U.S. counties between 1981–1983 and 2017–2019 time periods. In **b**, the 0th to 25th percentile (red) had percent decrease in cancer mortality of [−209.3, 6.2), the 26th to 50th percentile (orange) had percent decrease in cancer mortality of [6.2, 17.6), the 51th to 75th percentile (yellow) had percent decrease in cancer mortality of [17.6, 27.2), the 76th to 100th percentile (green) had percent decrease in cancer mortality of [27.2, 70.4].

Geographic distribution of cancer mortality improvement provides one window to address the question, “Who is benefiting from the decline in cancer mortality in the U.S.?”. In Fig. 2a, we mapped U.S. counties that have either improved their cancer mortality rates (depicted in green) or counties that have not experienced improvement (depicted in yellow). A visual inspection of the map reveals geographic patterns about the places in the U.S. that either improved or failed to improve during the period of dramatic national improvement in cancer mortality. The majority of the “no improvement counties” were in the central part of the nation. On the other hand, there was a strong pattern of cancer improvement in the northeast region, and along the coastlines of the Atlantic, Gulf of Mexico, and Pacific regions. While the majority of counties experienced improvement, there were a number of counties with rising cancer mortality. There were 460 counties with no improvement, and among them 458 counties were experiencing increased mortality rates.

To further investigate the magnitude of cancer mortality improvement we classified and mapped counties into percentiles (Fig. 2b). Counties depicted in green had the highest improvement followed by counties in yellow, orange, and red. The visual inspection of the percentile map reveals at least two patterns. First, counties with high level of improvements tend to be located in or near large metropolitan locations. The corridor from Boston to New York City (NYC) to Washington D.C. had concentrations of high improvement counties. Similar patterns were observed for South Florida (Miami), Southern California (Los Angeles and San Diego), the Bay area in Northern California (San Francisco), Colorado (Denver), and Southern Arizona (Phoenix). Second, the low percentile “red counties” with little or no decline in cancer mortality tended to be in the central part of the U.S. There were very few low percentile “red counties” located along the U.S. coast. This second pattern suggests that there is a coastal/inland component to the improvement in cancer mortality. An additional geographic visualisation (heat map) is provided as Supplemental Fig. 1. Similar geographic patterns in mortality have been examined previously for all cancers and specific types of cancer mortality [46].

### The level of income, urban status, and initial level of cancer mortality were associated with the degree of cancer mortality improvement for U.S. counties (between 1981–1983 and 2017–2019 time periods)

The equation below reports standardised estimates for our OLS model that predicts county-level percent decrease in cancer mortality rates between the 1981–1983 and 2017–2019 time periods (Supplemental Table 2). Estimates were significant ($$ < .05$$) with R^2^ = 0.65. We interpret parameters as county characteristics that indicate place-based benefits in reduction of cancer mortality net of other influences in the model. Place-based benefits of cancer mortality reduction were concentrated in counties with higher education, income, and urban population. The race association was relatively small and negative with a standardised estimate of -0.06. Interestingly, initial level of cancer mortality in 1981–1983 had a significant association with percent decrease over the study period extending to 2017–2019. It appears that counties with initially higher levels of cancer mortality had more room for improvement and, thus, were able to benefit more during a period of cancer mortality decline.

$$\hat{Y}=\alpha +.16{X}_{1}-.10{X}_{2}+-.06{X}_{3}+.31{X}_{4}+.32{X}_{5}$$

### U.S. counties with the highest income levels were the first to experience improved cancer mortality, the highest rates of improvement, and the greatest reduction in excess deaths

U.S. counties were grouped according to median household income, where approximately 10% of U.S. population were in each group (Supplemental Table 3). Group 1 consisted of counties with the highest median household income that collectively had approximately 10% of the nation’s population. Group 2 contained a group of counties that collectively had approximately 10% of the nation’s population and the next highest level of median income. Subsequent Groups 3 to10 were categorised accordingly, with Group 10 having the 10% of the population living in the lowest income counties. An examination of several metrics indicate how profoundly important income of place is associated with decline in cancer mortality rates. Clearly, high income places were major beneficiaries of national improvement in cancer mortality. High income counties were generally experiencing much higher levels of cancer mortality decline. The lowest income group (in comparison to the highest income group) had 10 times more counties with no improvement in cancer mortality, a 37% higher cancer mortality in 2017–2019, and one seventh the rate of improvement. Using the improvement rate of the highest income counties (Group 1) as the standard, we calculated excess deaths. This resulted in over 65,000 excess deaths in 2019 alone.

**Fig. 3:**
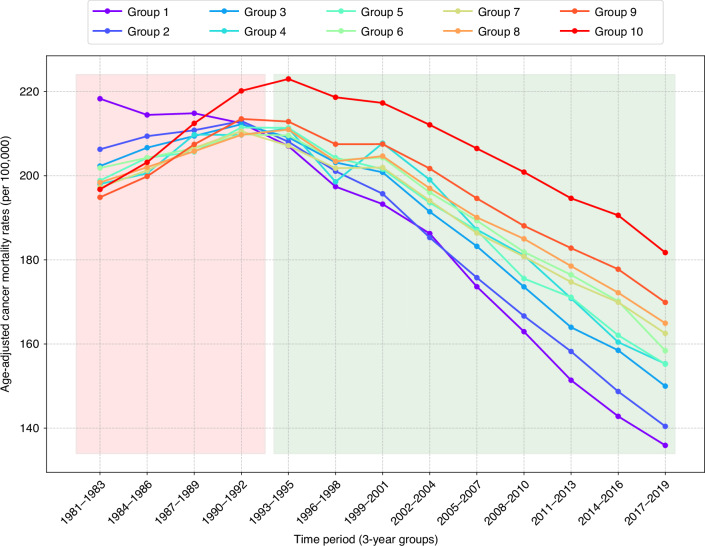
Trends of age-adjusted cancer mortality rates for U.S. counties grouped by median household income: 1981–2019. Each group of counties had approximately 10% of U.S. population. These groups are ordered from the highest income in Group 1 to the lowest income in Group 10. The red shaded box depicts a period of increasing cancer mortality and the green box the period of declining cancer mortality.

Figure 3 reveals a pattern of cancer mortality improvement where all income groups had declining cancer mortality rates, yet rates varied considerably among income groups. An initial period (1981–1983 to 1990–1992) of increasing rates of cancer mortality is depicted in the red shaded box. Then there was a sudden turnaround in cancer mortality in which rates began to drop. Generally, from 1993–1995 (green shaded box) cancer mortality began to decline for all income groups and a new pattern emerged. Income-based disparities became a feature of U.S. cancer mortality decline. Improvements were stronger among high income counties, indicating that income-based disparities in cancer mortality were increasing at the same time as cancer mortality was declining. High income counties in Group 1 were first to trend downward. Cancer mortality improvement was linked to income level of places, and as national level improvement in cancer mortality occurred, disparities were increasing. In summary, income-linked cancer mortality disparities were consistently growing as cancer mortality declined.

### OLS models based on income, education, urban, race, and initial cancer mortality became increasingly associated with improvement in cancer mortality as length of time series increased

**Fig. 4:**
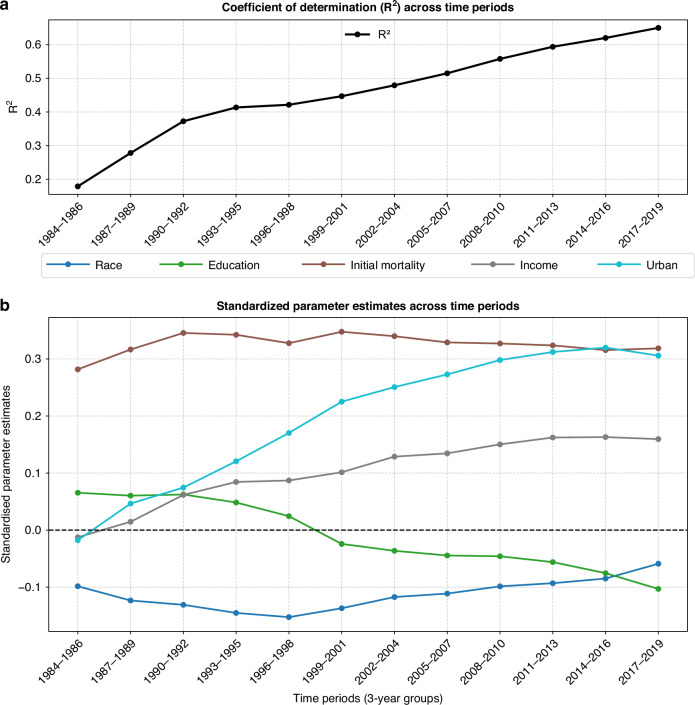
Parameter Outputs for 12 Time-ordered OLS Models of the Change in Age-adjusted Cancer Mortality Rates for U.S. Counties: 1981–1983 through 2017–2019. The parameter outputs for these 12 time periods include: **a** Coefficient of determination and **b** standardized parameter estimates.

Evaluation of models applied to 12 different periods provides more context for county-level improvements. First, the predictive power of models improved with additional years of data. The first-period model’s effectiveness was R^2^ = .18 and increased throughout the time series reaching R^2^ = .65 in 2017–2019 (Fig. 4a). Increasing levels of model power indicates that over time, measure of income, education, urban settings, and race were having a greater association with cancer mortality reduction.

Urban residence, higher income, and more education effects were becoming larger as the model was applied to additional years of data (Fig. 4b). Race (percent Black) had negative effects that decreased over time, approaching zero. This can be interpreted as declining racial disparities in cancer mortality improvements net effects of income, education, and urban residence [36, 57–61]. Initial mortality level (1981–1983) was included as a control variable to account for the possibility that initially high or low mortality levels were conducive to higher and lower rates of change. Standardised beta for initial mortality level ranged from 0.28 to 0.32 for the 12 model applications across time, indicating a consistent control factor that contributed to predictability.

An examination of the standard beta coefficients of the 12 time ordered OLS models reveals several significant patterns. As the selected time frame of the OLS models increased, urban residence became more associated with cancer improvement. By the end of the series, urban residence had the largest association with improving cancer mortality rates (beta = 0.31). Income levels of counties has similar patterns of increasing association with the beta coefficient of 0.16. Education levels of counties modestly declined across the time period. Race (percent Black) had a modest negative association with improving cancer rates, which tended to decline with each successive time period through 2017–2019 (beta = −0.06). The implication of these findings is that cancer improvement was substantially associated with urban and high income counties.

### Moran statistics identified concentrations of high rates of cancer mortality improvement in the Boston-NYC-Washington D.C. corridor and in urban centres on the California coast

**Fig. 5:**
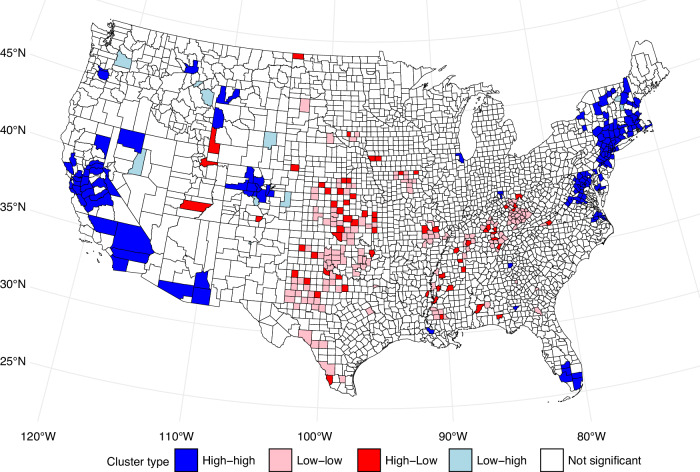
Local Moran’s I clustering on cancer mortality improvement between 1981–1983 and 2017–2019 time periods.

In Fig. 5, Local Moran’s I clusters of cancer mortality improvement, highlight areas of spatial autocorrelation. Counties marked in blue, represent regions with high cancer mortality decline adjacent to other high-improvement counties. These high–high clusters are primarily located in densely populated areas, often include major metropolitan areas. The majority of cancer mortality improvements are concentrated in a small number of large urban centres. High-improvement clusters are prevalent on the West Coast and the Boston-NYC-Washington D.C. corridor. The low-low clusters, depicted in pink, represent counties with minimal or no improvement, surrounded by similarly underperforming regions. These low-low clusters are concentrated in the South and Midwest, indicating areas where cancer mortality improvements are less pronounced.

### GWR Modelling suggested that the SDoH influences collectively were associated with cancer mortality decline nationwide while select individual social determinants varied by region

**Fig. 6:**
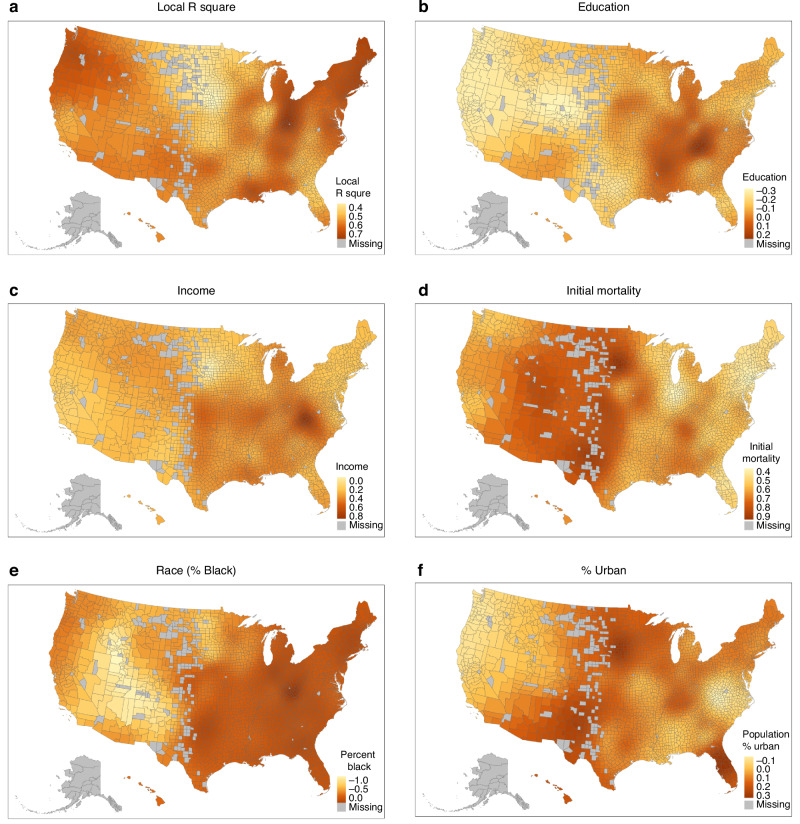
Geographic distribution of parameter outputs obtained from geographically weighted regression model with outcome as percent decrease in cancer mortality between 1981–1983 and 2017–2019 time periods. The reported model outputs consist of **a** R-squared values and regression coefficients for the following variables: **b** education, **c** income, **d** initial mortality, **e** race, and **f** percent urban.

Due to the evidence of spatial autocorrelation through Local Moran’s I clustering we extended our regression analysis to geographically weighted regression (GWR) model. The GWR results, therefore, complement the Local Moran’s I clusters, providing a more localised understanding of the association of the SDoH with cancer mortality trends. Results presented in Fig. 6 illustrate regional variations in factors influencing cancer mortality improvement across U.S. counties. The model’s explanatory power, as shown by the local R-square map, was moderately strong across the entire country ranging from 0.40 to 0.70 at the county level. It was strongest in the Northeast region, a band running from north Ohio/Michigan southward to the Gulf coast and the Pacific Northwest region. The association of higher levels of education tended to be positive in the Appalachian region and the region along the Mississippi River. Education effects were small or negative for much of the rest of the country. Median Household Income (MHI) association varied greatly, ranging from 0.00 to 0.80. The association of MHI was greatest in Appalachia and eastern interior of the nation. While there are substantial racial disparities in cancer mortality in cross-sectional data, the level of association of race is more complex for improvement in cancer mortality. In the Eastern half of the nation, where the majority of the Black Americans live, the association of race approached zero. In the Western portion of the country where there are very small Black populations, GWR models produce inverse estimates. Urban residence was most predictive of cancer mortality improvement in New England, the North Central region, the southern border states, and Florida. The initial mortality rate variable had a significant association for most locations ranging from 0.40 to 0.90. The greatest association was in areas of the Midwest and West Texas, where many counties have very small populations. The cancer mortality rates and rates of change in less populated counties are comparatively unstable. This instability can produce very high or very low rates of change and can have the consequence of producing a “regression to the mean effect”. This highlights the value of estimating initial mortality in our models. An implication of these GWR patterns is that not only are mortality improvement rates different across counties, there are also substantial geographic variations in the association of SDoH factors with mortality rate changes.

A cautionary note should be considered when evaluating these findings. Our primary focus is on the rate of change of AACM per 100,000 population. AACM rates are considered especially useful in comparative projects that contrast between places and overtime since they adjust the data to account for variations in population size and age structure. For our study between 1981–2019, we found a beginning national AACM rate of 209.5 deaths per 100,000 and an ending rate of 150.0 deaths per 100,000. The rate of change was a 28.4% decrease in AACM. This is an index of significant mortality decline and a quantitative estimate of improving national cancer health. But it is useful to put this in context of several related statistics. The total number of cancer deaths between 1981–2019 was over 22.2 million and the beginning level was approximately 428,000 in 1981 and increased each year to approximately 615,000 in 2019. That is, the number of cancer deaths increased 30.4% between 1981 and 2019 while the AACM rate declined (−28.4%). This apparent discrepancy is a result of rapid growth and aging of the U.S. population; two influences that were removed by the AACM calculations. Implications of taking both estimates into account is far reaching. The declining AACM rate tells us that the U.S. is having success addressing cancer. Yet, the increasing total number of cancer deaths indicates growing demand for cancer health services and interventions.

## Discussion and conclusions

We asked the question: “Who is benefiting from the decline in cancer mortality in the United States?” By examining 39 years of change in cancer mortality for 2954 counties, we have a partial answer. Most of the population lived in counties that were experiencing improvement in cancer mortality. This represents a significant turnaround in the trajectory of the nation’s health; a transition from increasing to declining cancer mortality. Our study builds on and extends existing research by documenting large geographic differences in mortality improvement at the county level. It suggests that certain geographic locations and certain SDoH factors were associated with higher reduction in cancer mortality. Metropolitan areas, coastal settings, and higher income locations were experiencing the greatest improvements.

We conducted a GWR analysis to explore the utility of our OLS modelling from the point of view of each county. The results of this analysis indicated that the association of SDoH influences with improvement of cancer mortality was pervasive throughout the nation with power ratings ranging generally from 0.40 to 0.70. However, specific SDoH influences tended to have stronger associations in specific regions of the country. Income associations with cancer decline was strongest in the Appalachian region and the eastern half of the country. Urban association with cancer decline was stronger in New England, the North Central region, the southern border states, and Florida. These findings call for additional studies to understand the regional dynamics of SDoH influences.

Our series of twelve OLS models indicated that the larger reductions in cancer mortality were concentrated in urban and high-income counties and that the strength of these SDoH factors were systematically increasing with the length of the timespan. Following the SDoH perspective, we hypothesise that counties with more favourable SDoH factors would more likely encourage and facilitate the adoption of health practices that protect against cancer. Counties with higher income and urban status would adopt innovations that decreased cancer mortality earlier and at much higher rates than other counties. For example, it follows that such tobacco control innovations as smoking cessation programmes, adoption of smoke-free laws, higher tobacco taxes, labelling of risk on tobacco products, and limiting media glamorisation of smoking would be adopted more frequently and accumulating at higher rates in urban and higher-income counties. One could also surmise that there were higher levels of adoption practices for cancer screening, cancer treatments, healthier diets, and exercise in favourable SDoH settings.

At the same time metropolitan and high-income counties were experiencing rapidly improving cancer mortality, rural and low-income counties were experiencing lower levels of improvement. This pattern resulted in growing disparities between urban and rural places and high- and low-income areas. The time series data reveals the growth of SDoH-linked disparities as the overall improvement of cancer mortality progressed. This linkage between health improvement and growing disparities may be a frequent aspect of advances in the nation’s health. We speculate that health innovations are rarely implemented equitably throughout society and consequently can be, at the same time, the source of both health improvements and new disparities. From this perspective urban and high-income locations are more often the places creating and adopting innovations including cancer health innovations and thus were experiencing the greatest reductions in cancer mortality. It appears that as a nation we have been better at innovating approaches to cancer health than in diffusing these innovations. As cancer mortality was improving, large place-based disparities were emerging; the level of benefits from the national reduction in cancer were hardly being shared widely and equitably. This paradoxical linkage between successful approaches to health improvement and the creation of new disparities associated with lower levels of diffusion is a topic worthy of further studies. The profound variations in cancer mortality trends have implications for clinical practice, public health, policy development, resource allocation, and cancer research. We acknowledge that there are several limitations of the study, and they are provided in supplemental information. This study provides an argument for incorporating place-based strategies in developing initiatives designed to advance cancer health and more equitable cancer mortality outcomes.

## Supplementary information

Supplementary Information

## Data Availability

The data used in this study is open-source and available online provided by the National Centre for Health Statistics, Centres for Disease Control and Prevention, U.S. Census, and the Economic Research Service, USDA (see Supplemental Table 1).
