# Supplementary material for: Who is benefiting from the dramatic decline in U.S. cancer mortality? Place-based evidence of disparities in rates of improvement
Source: Br J Cancer. 2026 Mar 30;134(10):1468–78. doi: 10.1038/s41416-026-03339-8 (PMC13133269; doi:10.1038/s41416-026-03339-8)
Supplement: Supplementary file 1 — Supplementary Information [file 41416_2026_3339_MOESM1_ESM.docx]

| **Variable** | **Year** | **Website** |
| --- | --- | --- |
| Age-adjusted Cancer Mortality Rates (Neoplasms) | 1981 to 2016 (3 years as a group) | <https://wonder.cdc.gov/> |
| Age-adjusted Cancer Mortality Rates (Neoplasms) | 2017 to 2019 (3 years as a group) | <https://wonder.cdc.gov/ucd-icd10.html> |
| Race (Percent Black Population) | Annually from 1981 to 2019 | <https://seer.cancer.gov/popdata/download.html#single> |
| Median Household Income | 1979, 1989, 1999 | <https://www.census.gov/data/tables/time-series/dec/historical-income-counties.html> |
|  | 2009 to 2019 | <https://www.ahrq.gov/sdoh/data-analytics/sdoh-data.html> |
| Education | 1980, 1990, 2000, 2014-2018 combined | <https://www.ers.usda.gov/data-products/county-level-data-sets/county-level-data-sets-download-data/> |
| Percent Rural | 2000 | <https://data.census.gov/table/DECENNIALSF32000.H005?q=population%20percent%20rural%20by%20county%20in%202000> |
|  | 2010 | <https://www.census.gov/programs-surveys/geography/guidance/geo-areas/urban-rural/2010-urban-rural.html> |
|  | 2020 | <https://www.census.gov/programs-surveys/geography/guidance/geo-areas/urban-rural.html> |

Supplemental Table 1: Data sources.


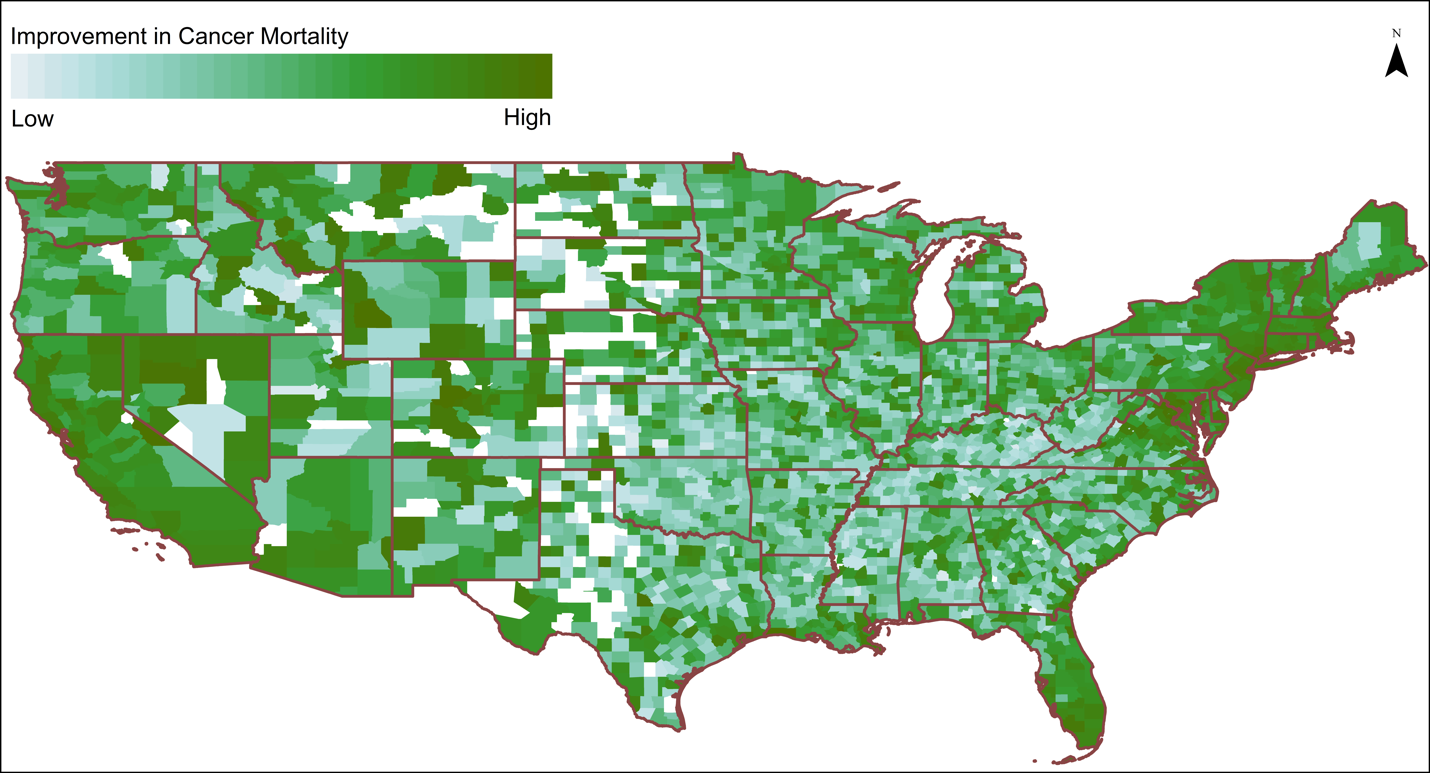


Supplemental Figure 1. Heat Map of County-level Improvement in Age-adjusted Cancer Mortality Rates in U.S. Counties: 1981-1983 through 2017-2019.

*Note.* Improvement in age-adjusted cancer mortality was calculated as the percent decrease in age-adjusted cancer mortality rates between 1981-1983 and 2017-2019. This visualization is restricted to data from contiguous states.

| **Variable** | **Standardized Estimate** | **Standard Error** | **t Value** | **Pr (> \|t\|)** |
| --- | --- | --- | --- | --- |
| **Intercept** | 0.01293 | 0.01358 | 0.95211 | 0.34112 |
| **Percent Urban** | 0.30593 | 0.01066 | 28.70729 | 0.00000 |
| **Education** | -0.10314 | 0.00853 | -12.09826 | 0.00000 |
| **Median Household Income (MHI)** | 0.15949 | 0.00711 | 22.44144 | 0.00000 |
| **Percent Black (Race)** | -0.05902 | 0.00999 | -5.90799 | 0.00000 |
| **Initial Mortality Rate (1981-1983)** | 0.31862 | 0.01078 | 29.55689 | 0.00000 |

*Note.* Dependent variable for the OLS model was percent decrease in age-adjusted cancer mortality for 2,954 U.S. counties between 1981-1983 and 2017-2019 time periods. The model was weighted by county population size. The overall predictive power was estimated as R^2^= 0.65. All the numbers in the table are rounded to five decimal places.

Supplemental Table 2. Parameter Estimates of Age-adjusted Cancer Mortality Decline Model (OLS regression) in U.S. Counties.

Supplemental Table 3. Comparison of County Income Levels and Decline in Age-adjusted Cancer Mortality in the United States: 1981-1983 through 2017-2019

| **Income Group of U.S. Counties** | **Combined Population 2019** | **Median Household Income in Dollars 2019** | **Percent Counties with No Improvement or Increasing Cancer Mortality** | **Cancer Deaths 2019** | **Excess Cancer Deaths 2019** | **Cancer Mortality**  **1981-1983** | **Cancer Mortality**  **2017-2019** | **Percent Decrease in Cancer Mortality 1981-2019** |
| --- | --- | --- | --- | --- | --- | --- | --- | --- |
| **10** | **32764805** | **42246.26** | **33.2** | **60216** | **14780** | **197.73** | **183.78** | **5.47** |
| **9** | **32791994** | **51059.34** | **19.42** | **56890** | **11454** | **198.43** | **173.49** | **10.87** |
| **8** | **32723227** | **55424.35** | **14.32** | **54251** | **8815** | **194.66** | **165.79** | **13.16** |
| **7** | **31692447** | **59515.23** | **8.84** | **51199** | **5762** | **199.8** | **161.55** | **17.93** |
| **6** | **32983096** | **63285.54** | **9.57** | **51395** | **5959** | **196.75** | **155.82** | **19.69** |
| **5** | **32709327** | **67104.31** | **9.09** | **51336** | **5900** | **199.38** | **156.95** | **19.54** |
| **4** | **33526606** | **71227.35** | **12.37** | **51347** | **5910** | **192.26** | **153.15** | **18.34** |
| **3** | **32839771** | **77258.99** | **4.55** | **48904** | **3468** | **201.76** | **148.92** | **24.6** |
| **2** | **34414970** | **88401.29** | **2.11** | **48960** | **3524** | **204.79** | **142.26** | **29.58** |
| **1** | **33810881** | **108392.4** | **3.03** | **45436** | **-** | **216.7** | **134.38** | **36.7** |
| **Total/**  **Mean** | **330257124** | **68391.5** | **11.65** | **519934** | **65572** | **200.23** | **157.61** | **19.59** |

*Note.* Metrics included: the average of counties’ median household income in 2019; percent of counties within each group that had no improvement in age-adjusted cancer mortality rates; estimated total age-adjusted deaths for each group from cancer in 2019; estimated excessed deaths assuming all groups had the mortality improvements as high income group (Group 1); average age-adjusted cancer mortality rates of counties in each group for 1981-1983 and 2017-2019 periods; and average percent change in cancer mortality of counties in each group from 1981 to 2019.

Supplemental Figure 2. Trends in Age-adjusted Cancer Mortality Rates for Low Income Urban Counties, Low Income Rural Counties, High Income Urban Counties, and High Income Rural Counties for U.S.: 1981-1983 through 2017-2019.

*Note.* Counties were classified as urban utilizing the 2013 U.S. Office of Management and Budget (OMB) Metropolitan Area Delineation. Counties not classified as metropolitan were used as an indicator of rural. Counties with greater than average income (median household income) were classified as high income. Counties with below or equal to average income (median household income) were classified as low income.


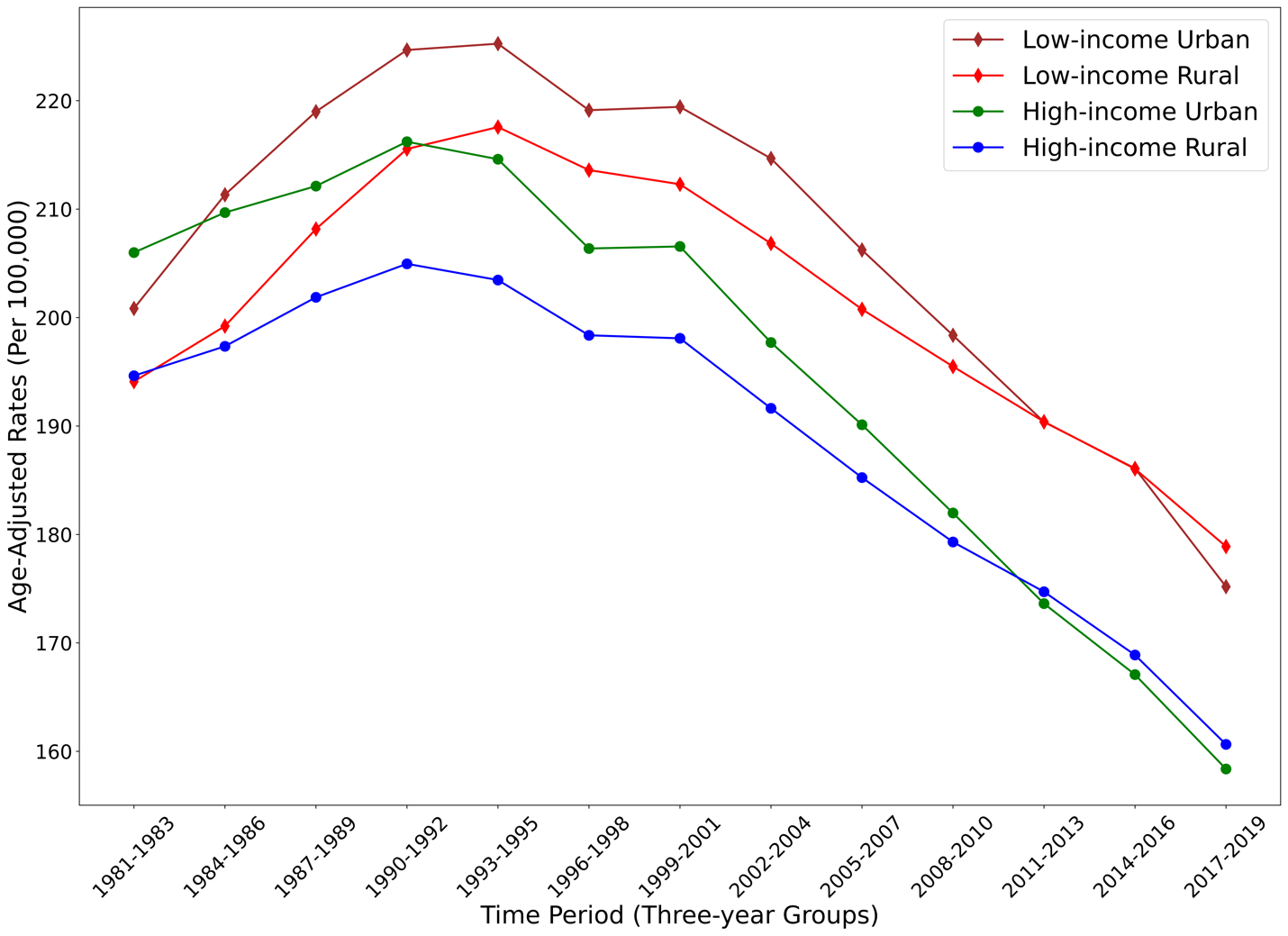


Limitations of the study

There are several limitations that should be considered in interpreting the results of our analysis.

1. To improve the estimation of counties with small populations, we aggregated county mortality rates into three-year segments and consequently our temporal resolution is for 3-year segments rather than 1-year segments.
2. The selection of starting time period can greatly influence the magnitude and pattern of change. We intentionally selected the 1981-1983 period as a starting point because the time frame from 1981 to 2019 captures three important phases of cancer mortality: (1) a period of increasing mortality of the nation, (2) the peak level of national cancer mortality and then (3) the period of cancer mortality decline. Selection of any other time frame can result in different improvement estimates.
3. Even with the three-year aggregated mortality rates there is not sufficient deaths in many counties to estimate mortality improvement by gender, age, and race/ethnicity groups.
4. To answer the question “Who is benefiting from the decline in cancer mortality in the U.S.?”. In this study we have selected a set of analytical procedures that include visual mapping of improvement, OLS regression, Local Moran’s I clustering, and geographically weighted regression (GWR) to provide important insights about cancer mortality improvement at the county level. However, there are several other modeling dimensions that can be used such as multilevel and spatial machine learning models that can provide additional insights into the research question. For example, OLS and GWR models struggle to capture multi collinearity among the input variables and may not efficiently handle non-linear relationships between input and output variables. So, in future research, we suggest considering alternate models such as geographically weighted Random Forest models to overcome the limitations.
5. Our unit of analysis is US counties, which has the advantage of having numerous governments collected indicators available across counties and overtime. There is a serious limitation concerning the scale of the various counties. For example, Los Angeles County in California has a 2020 census population of about 10 million citizens, yet we treat it in our analysis at the same level as a rural county of 2000 citizens. We clearly cannot capture important variations in the levels of cancer improvements between neighborhoods, cities, or other subdivisions within Los Angeles County.
6. Another limitation involves the tradeoffs of model selections and analytical questions addressed. For example, we estimate rates of improvements in cancer mortality at the county level with respect to initial mortality rates, income levels, education levels, percent Black, and percent rural. There are certainly many other possibly county characteristics that could and should be analyzed in additional research. Even more interestingly, we did not investigate the relative acceleration of improvement at different places and under different conditions.
